# Base Dynamics in the *Hha*I Protein Binding Site

**DOI:** 10.1021/acs.jpcb.3c03687

**Published:** 2023-08-10

**Authors:** Kari Pederson, Gary A. Meints, Gary P. Drobny

**Affiliations:** † Department of Chemistry & Biochemistry, California State University at Dominguez Hills, Carson, California 90747, United States; ‡ Department of Chemistry, Missouri State University, Springfield, Missouri 65897, United States; § Department of Chemistry, University of Washington, Seattle, Washington 98195-1700, United States

## Abstract

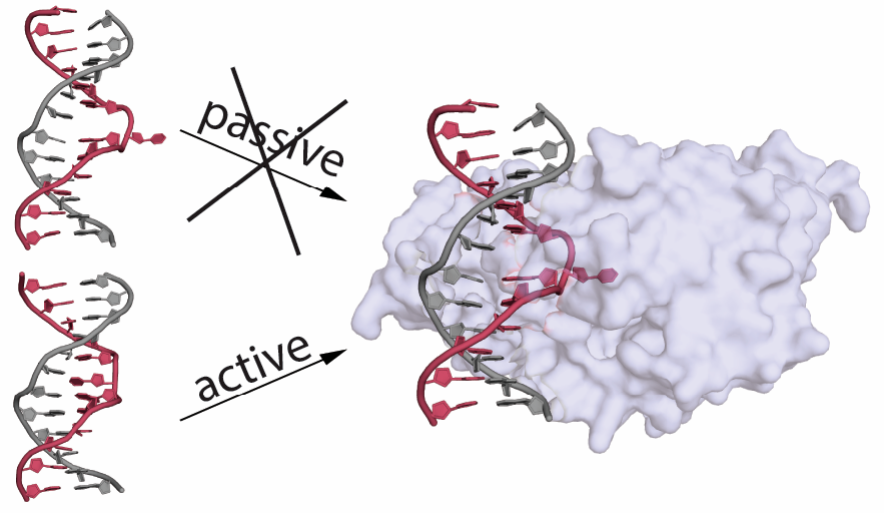

Protein–DNA interactions play an important role in numerous biological functions within the living cell. In many of these interactions, the DNA helix is significantly distorted upon protein–DNA complex formation. The *Hha*I restriction-modification system is one such system, where the methylation target is flipped out of the helix when bound to the methyltransferase. However, the base flipping mechanism is not well understood. The dynamics of the binding site of the *Hha*I methyltransferase and endonuclease (underlined) within the DNA oligomer [d(G_1_A_2_T_3_A_4_G_5_C_6_G_7_C_8_T_9_A_10_T_11_C_12_)]_2_ are studied using deuterium solid-state NMR (SSNMR). SSNMR spectra obtained from DNAs deuterated on the base of nucleotides within and flanking the [5′-GCGC-3′]_2_ sequence indicate that all of these positions are structurally flexible. Previously, conformational flexibility within the phosphodiester backbone and furanose ring within the target sequence has been observed and hypothesized to play a role in the distortion mechanism. However, whether that distortion was occurring through an active or passive mechanism remained unclear. These NMR data demonstrate that although the [5′-GCGC-3′]_2_ sequence is dynamic, the target cytosine is not passively flipping out of the double-helix on the millisecond–picosecond time scale. Additionally, although previous studies have shown that both the furanose ring and phosphodiester backbone experience a change in dynamics upon methylation, which may play a role in recognition and cleavage by the endonuclease, our observations here indicate that methylation has no effect on the dynamics of the base itself.

## 1 Introduction

DNA methylation is considered to be vital for normal cellular development. CpG dinucleotide methylation has significant consequences in eukaryotes. Methylation has been shown to reduce the binding efficiency of many transcription factors^1−3^ and is implicated in gene silencing in eukaryotes either through a direct interference with transcription factor binding or an indirect attraction of proteins which have a high affinity for methylated DNA.^4^ For example, X-chromosome inactivation is posited to result from methylation-induced structural and/or dynamical changes within a triplet of CpG dinucleotides that then alters binding of the histone octamer through translational positioning of nucleosomes.^5−9^ In particular, methylated CpG dinucleotides are also sites for a high percentage of point mutations,^10^ and abnormal methylation patterns in DNA have been linked to various cancers^11−13^ and genetic disorders, such as fragile X, Beckwith–Wiedemann, Prader–Willi, and Angelman syndromes.^1−3,14^

**Figure 1:**
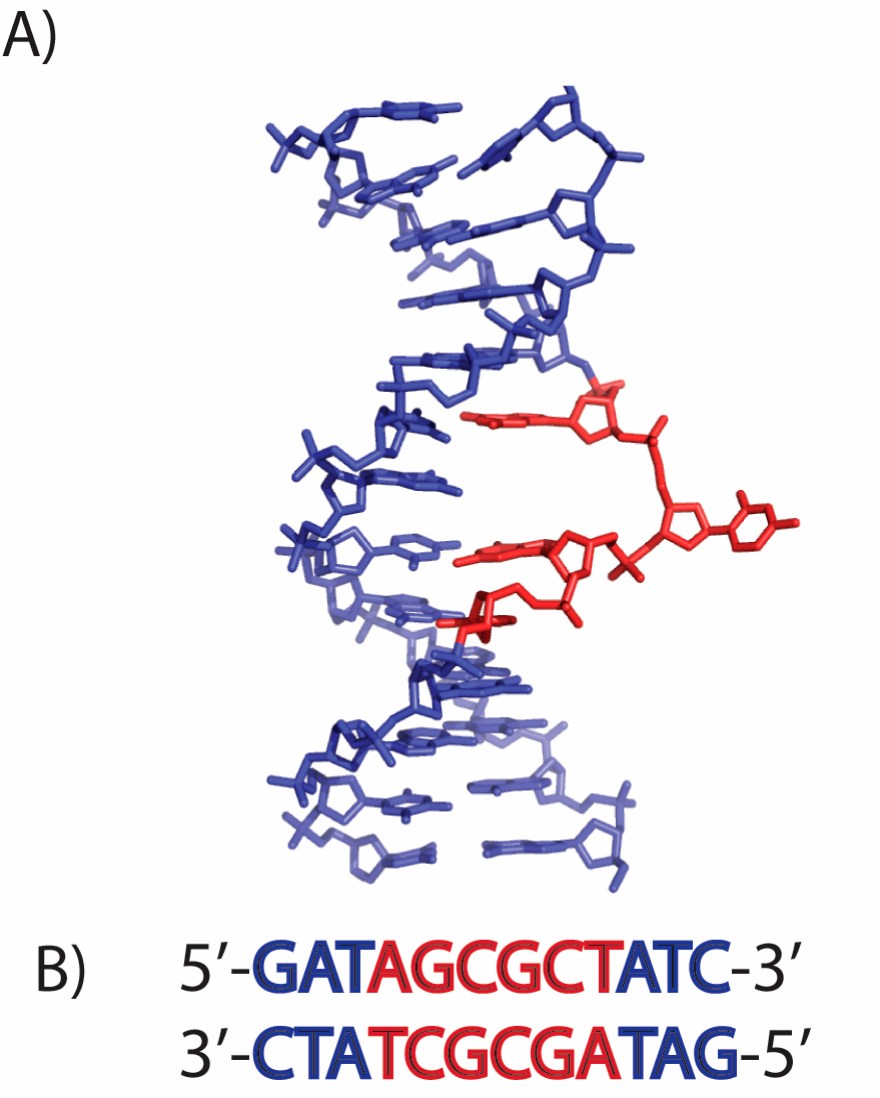
(A) *Hha*I target DNA, shown in red, showing the flipped deoxycytidine observed in the protein–DNA complex (protein removed). (B) Sequence of the target DNA; red highlighting indicates residues labeled with ^2^H on the base within and flanking the recognition sequence.

In prokaryotic cells, DNA methylation is primarily employed by restriction-modification systems that serve to prevent phage infection. In these systems, methylation is performed by three distinct types of methyltranferases (Mtases),^15,16^ of which the simplest are the type II Mtases that are cognate to the well-known restriction endonucleases.^17,18^ The *Hha*I system is one such system, where both the endonuclease and methyltransferase recognize the sequence [5′-GCG↓C-3′]_2_ (where the cleavage site is indicated by the arrow and the methylation target is underlined). The crystal structure of the *Hha*I Mtase bound to its cognate DNA with the *S*-adenosyl-methionine (Ado-Met) cofactor shows that the cytosine has been flipped out of the double helix and is surrounded in the binding pocket of the Mtase (Figure 1A).^19^ This base flipping provides a pathway for methylation of the base to take place; however, it is still unclear whether the base is actively flipped out by the protein or passively captured as it rotates out of the helix. Early computational and NMR imino proton exchange studies supported passive flipping;^20,21^ however, active flipping^22−29^ through the major groove pathway^30−34^ is supported by computational and mutational studies from the past two decades.

The central base pairs of alternating GCGC sequences have been shown to experience lifetimes which are shorter than isolated GC base pairs but longer than other GC base pairs within a GC tract.^21^ Additionally, computational studies of nucleotide stacking interactions demonstrated that the GC/GC step is the most stable followed by CG/CG.^35^ When these dinucleotide steps are overlapped, as in the [5′-GCGC-3′] site, the target C experiences the most unfavorable interstrand interactions, while the base pairing G has a strongly favorable interaction with the corresponding G from the complementary strand,^35^ which may help with stabilization of the orphaned G during base flipping. Structural and binding studies of the *Hha*I Mtase–DNA complex with a mismatched or abasic target base pair demonstrate that flipping still occurs and that the DNA is more tightly bound.^32,36,37^ Furthermore, base flipping has been shown to occur even in the absence of the catalytic loop.^38^ This strongly suggests that (1) the primary recognition mechanism between the protein and the DNA occurs between the protein and the phosphodiester backbone of the DNA, (2) that the Mtase may overcome base pairing interactions to stabilize the open state of the base pair as has been observed for uracil DNA glycosylase (UDG),^39^ and (3) that base flipping and methylation are not coupled.^40^

The importance of understanding the base flipping mechanism has been made even more evident by recent studies of the catalytic process indicating that base flipping and not catalytic loop closing is the rate-limiting step for methylation.^41,42^ Although the recognition process between the Mtase and the methylation target has been well studied^24,28,34,38,40,43−48^ and the methylation mechanism has recently been modeled,^49,50^ there has been limited quantitative analysis of the effect of methylation on the base of the target site in this sequence and very few studies on the stability of the target base pair.^21^ Solid-state NMR (SSNMR) analysis of the motion of the backbone and furanose rings of the nucleotides within and flanking the recognition sequence of the *Hha*I methyltransferase target DNA has shown that while the identity of the residue (2′-deoxycytidine) plays the most significant role in furanose ring dynamics, the backbone dynamics are strongly influenced by the sequence position.^47,51^ The combined result of these factors leads to the methylation target experiencing large amplitude dynamics in both moieties.

We have previously suggested that the *Hha*I target sequence may exhibit sequence-specific motions that facilitate base flipping by reducing energetic barriers and that changes in orientation of the backbone upon methylation may be important for enzyme recognition.^47,51,52^ The present study represents the final installment of this work, investigating the dynamics of the bases within the recognition site by reporting SSNMR line shape and relaxation data and modeling the deuterium solid-state line shapes and relaxation times as a function of the trajectories of the base deuterons in the protein recognition site. Limited conformational flexibility is observed in the base of the target site, which is unaffected by methylation, suggesting a mechanism through which dynamics of the backbone may assist the Mtase in discerning the target and nontarget C bases within the recognition site and then flipping is achieved through an active mechanism.

## 2 Methods

### 2.1 Sample Preparation

**Figure 2:**
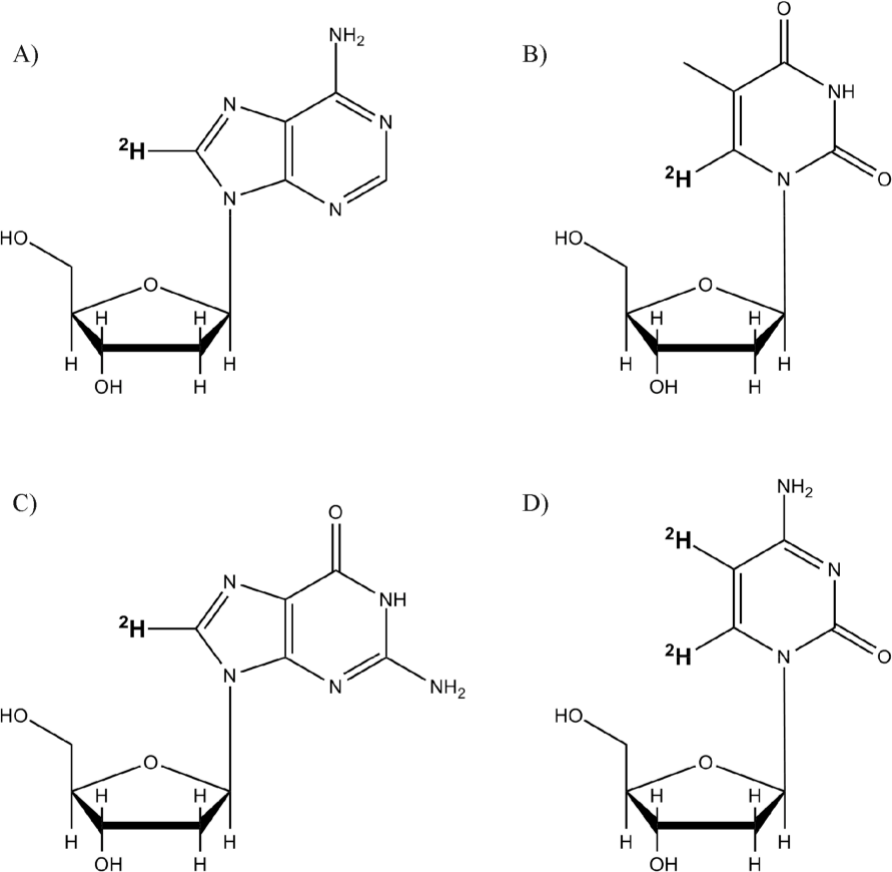
Specific labeling of the bases of A) 2′-deoxyadenosine, B) 2′-deoxyguanosine, C) 2′-thymidine, and D) 2′-deoxycytidine, where the 5-methyl-cytosine replaced the ^2^H atom at the 5 position of the cytosine ring with an unlabeled methyl group.

Deuterium labels were incorporated into the base at positions A4, G5, C6, G7, C8, T9, and 5-methyl-C6 in [d(G_1_A_2_T_3_A_4_G_5_C_6_G_7_C_8_T_9_A_10_T_11_C_12_)]_2_ (Figures 1B and 2). [5,6-^2^H]-2′-dC, [6-^2^H]-2′-dT, and [8-^2^H]-2′-dA were synthesized by the methods of Huang et al.^53^ The starting material for each synthesis was the 2′-deoxynucleoside, purchased from Berry & Associates. Phosphoramidites of deuterated nucleosides were synthesized by Glen Research, and [8-^2^H]-2′-dG-CE phosphoramidite was purchased from Glen Research. Oligonucleotides were synthesized and purified by TriLink BioTechnologies and prepared for NMR studies by salting, packing, and hydrating as has been described previously.^47,51^

### 2.2 SSNMR Spectroscopy

All experiments were performed on a Bruker NMR spectrometer, operating at a deuterium Larmor frequency of 76.776 MHz, corresponding to a magnetic field strength of 11.74 T, as has been described previously.^47,51^ Typical experimental times were 48 h per line shape, or ∼160000 scans. Variations in signal-to-noise were a result of differences in sample size. ⟨*T*_1*Z*_⟩ and ⟨*T*_1*Q*_⟩ relaxation times were measured using traditional inversion recovery^54^ and quadrupolar inversion recovery^55^ pulse sequences, with composite 180° and 90° pulses, respectively. Data for the [5,6-^2^H]-C6 was collected much earlier in the study, and the sample was unavailable for ⟨*T*_1*Q*_⟩ data collection.

In all experimental spectra shown, the line shapes have been symmetrized by averaging the points equidistant from the center of the spectrum to remove any artifacts from imperfect pulses; potential small contributions from the CSA were assumed to be negligible. The center isotropic peak is due to residual HDO.

### 2.3 Line Shape and Relaxation Analysis

**Figure 3:**
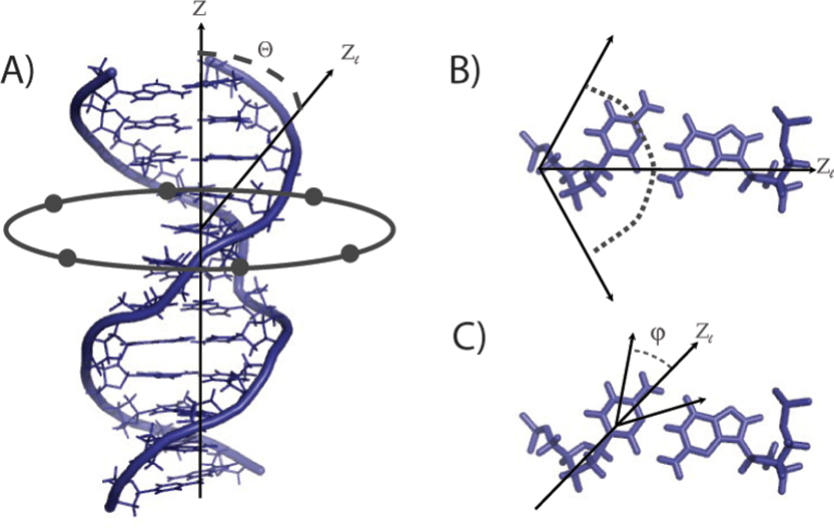
Line shape and relaxation simulations of the bases include three independent motions. (A) Slow (rate of 10^4^ Hz) uniform rotation of the DNA helix occurs about the helical symmetry axis, labeled *Z*. Local motion of the base is referenced to a local coordinate system, where the *z* axis is indicated by the vector *Z*_local_ defined by the angle Θ. The faster (rate 10^6^–10^9^ Hz) local motions of the base are represented by (B) a breathing motion shown by arclike trajectories and (C) small base librations for the bases of each nucleotide.

Simulated line shapes and relaxation times were calculated from parameters describing the global and local motions (Figure 3) using the program MXET1 developed by the Vold group.^56^ The global motion has been previously well-described using a six site diffusion on a cone.^47,51^ Here the half angle of the cone, describing the orientation of the local dynamic axis of the C–^2^H bond with respect to the longitudinal helix axis, was fixed at 90° to reflect the average orientation of the DNA base.

Imino proton exchange studies have long shown that DNA undergoes a “breathing” motion.^57−61^ The rates of base-pair opening have been shown to be sequence-dependent^62,63^ and are especially high in GC tracts.^21^ In this motion, the base is considered to be undergoing a simple excursion in and out of the helix, where the angle ϕ gives the amplitude of the excursion. This simple motion can be described by a single-well restoring potential of the form
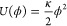
1where the force constant is κ. An additional small base libration motion, modeled as a two-site jump, was included to improve fits to ⟨*T*_1*Z*_⟩ and ⟨*T*_1*Q*_⟩ relaxation times but did not significantly affect the line shape.

#### 2.3.1 ^2^H Line Shape Calculations

The general formalism for modeling the ^2^H conformational dynamics have been described in detail previously.^47,51^ To obtain a ^2^H simulated line shape, the ω and π matrices must be determined. These matrices describe the trajectory that the C–^2^H bond follows and the nature of the energy surface that C–^2^H encounters as it travels along the trajectory, respectively.

As described above, the trajectory (ω) here is described as a combination of a six site jump diffusion on a cone and a ten site diffusion through a single-well restoring potential to represent the global helical rotation and DNA breathing motions, respectively. The half angle of the cone was set to 90°, and the helical rate of rotation was set to 10^4^ Hz. The number of sites and rate of the helical motion have been previously established as sufficient for ^2^H line shapes acquired at hydration levels greater than or equal to *W* = 10.^47,51,52,64^ Ten sites have been shown to be sufficient to effectively simulate a continuous process such as the breathing motion modeled here.^65,66^

The exchange matrix (π) was constructed as described previously to model the breathing motion.^47^ For the Hookean-well potential function of the form given in eq 1, ϕ is discretized to the set of angles {ϕ_*i*_}, and *i* labels the site along the discretized trajectory, and κ is the restoring force constant. For the ten site model used here, . Assuming a given potential *U*, the line shapes are simulated as a function of κ, *D*, and the angular displacement trajectory of the base.

#### 2.3.2 Partially Relaxed Line Shapes

In order to simulate ⟨*T*_1*Z*_⟩ and ⟨*T*_1*Q*_⟩ relaxation times, it is necessary to calculate the spectral density function. The correlation function for a system with two intermediate frames is given by^67^
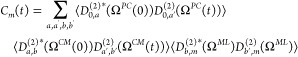
2

The correlation function can be simplified through careful choice of the axis system and powder-averaging as has been described previously and gives^67^
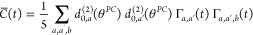
3

The two-axis motion used in the analysis of the bases is simplified by the use of a discrete-jump motion for the base libration. The correlation function for the base libration motion is given by^67^
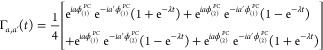
4where the subscript indices next to ϕ^*PC*^ are the site indices. Here, ϕ_(1)_^*PC*^ = −ϕ_(2)_^*PC*^ = ϕ^*PC*^, and eq 4 is simplified as
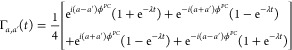
5

The second-axis motion is modeled as a diffusive motion in a restoring potential of the form given in eq 1 with ϕ = ϕ^*CM*^. In this case, the probability of a bond being distributed at ϕ^*CM*^ at time *t* is obtained by solving the diffusion equation
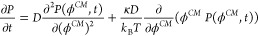
6

Given the initial condition, *P*(ϕ^*CM*^,0) = δ(ϕ^*CM*^ – ϕ_0_^*CM*^), eq 6 has the well-known solution^68^
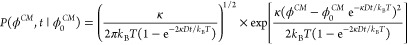
7where the a priori probability *W*(ϕ^*CM*^) is
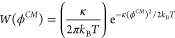
8

Because the relaxation of the base deuterons is affected by both the diffusive motion and the base libration, the correlation function triad for this motion is given by
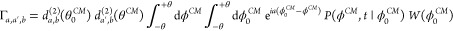
9

Substituting into eq 3 gives the following form of the powder-averaged correlation function
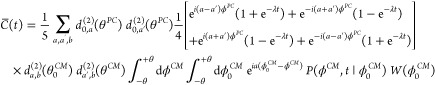
10where θ_0_^*CM*^ = θ^*CM*^ = 0 and
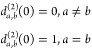
11allowing eq 10 to simplify to
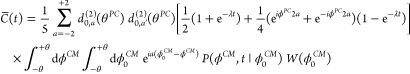
12

Furthermore, for  as defined for the bases
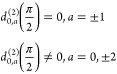
13which gives the following expression for the powder averaged correlation function
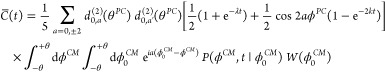
14

By substitution, the expression for the probability can be written as a series of Hermite polynomials^67,69,70^
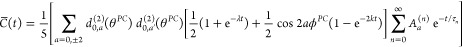
15or
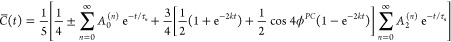
16where
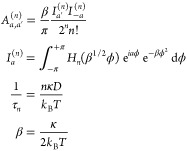
17and the resulting spectral density is
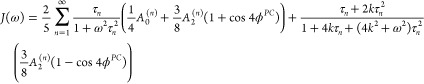
18

The powder-averaged spin–lattice relaxation rates for the Zeeman and quadrupolar order, respectively, are
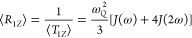
19
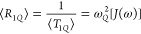
20

## 3 Results

**Figure 4:**
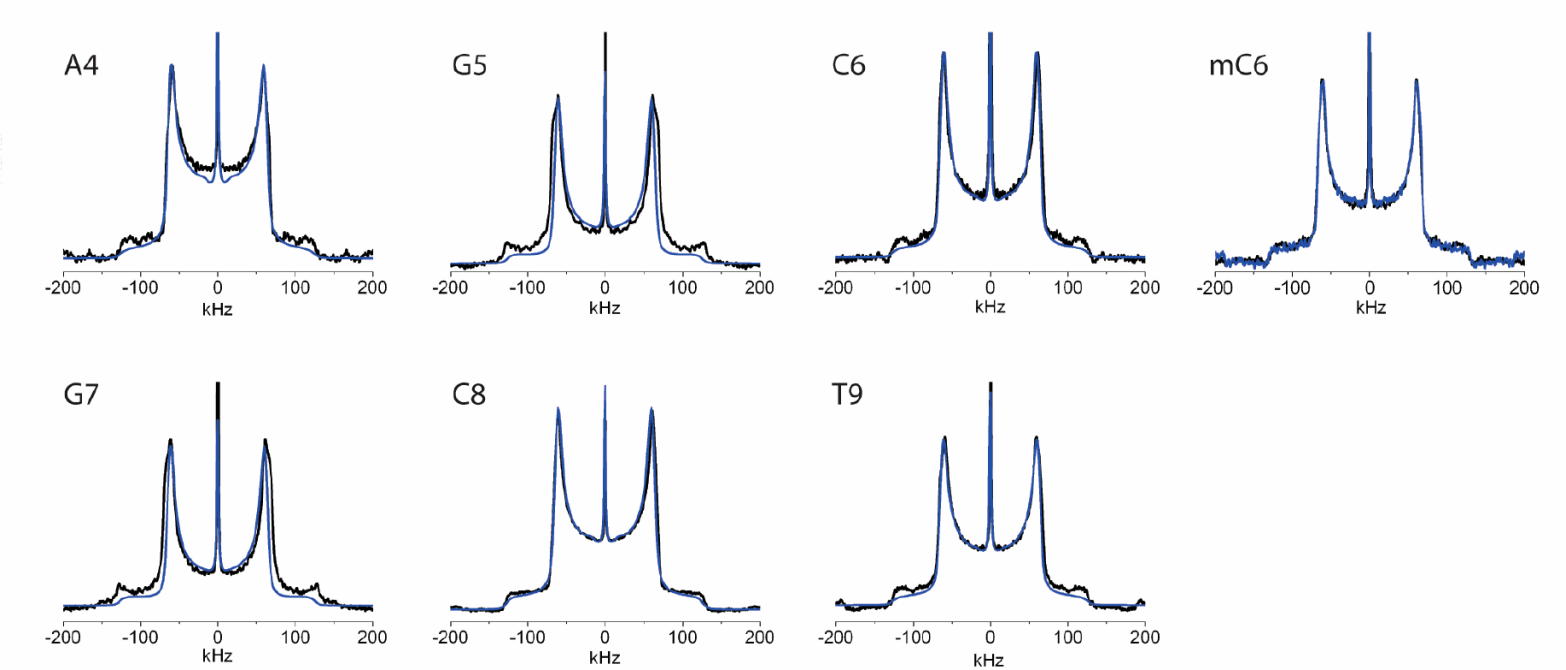
Seven deuterium line shapes (black) for each of the labeled sites in the nonmethylated and methylated DNA dodecamer with the simulation (blue) of each overlaid. The intensity of the simulated spectra was scaled so that the maximum height of the horns matched that of the experimental spectra. Simulation parameters are described in Table 3.

To probe the local mobility of the bases within and flanking the d(G_5_C_6_G_7_C_8_) site, deuterium line shapes and spin–lattice relaxation rates were obtained for the base deuterons of A4, G5, C6, G7, C8, T9, and 5-methyl-C6. Experimental line shapes and line shape simulations (sensitive to motions on the millisecond–nanosecond time scale) are shown in Figure 4. Spin–lattice relaxation rates are sensitive to motions ranging from picoseconds to microseconds, which are calculated from partially relaxed line shapes and given in Table 1.

**Table 1:** Experimental and Simulated Spin–Lattice Relaxation Times for Base-Labeled A4-T9 from the DNA Sequence [d(GATAGCGCTATC)]_2_

		⟨*T*_1*Z*_⟩ (ms)		⟨*T*_1*Q*_⟩ (ms)	
sample (from [d(GATA**GCGC**TATC)]_2_)	hydration (waters/nucleotide)	experimental	simulated	experimental	simulated
[8-^2^H]-A4	10.4 ± 1	105 ± 4	124	115 ± 6	125
[8-^2^H]-G5	9.7 ± 1	151 ± 7	160	136 ± 11	117
[5,6-^2^H]-C6	11.1 ± 1	144 ± 7	153		
[8-^2^H]-G7	10.0 ± 1	147 ± 5	160	160 ± 17	117
[5,6-^2^H]-C8	10.5 ± 1	244 ± 18	256	195 ± 20	218
[6-^2^H]-T9	10.3 ± 1	153 ± 6	161	116 ± 6	137
[6-^2^H]-5-methyl-C6	9.5 ± 1	150 ± 5	153	158 ± 8	143

As has been described previously, motions occurring in the millisecond–nanosecond range produce “intermediately averaged” line shapes of the type shown in Figure 4.^47,66^ The features of these line shapes can be measured to aid in a comparison of line shapes. Quantitatively, the width between the horns is 3/4 of the effective quadrupolar coupling constant (QCC_eff_) and the amplitude reduction factor (ARF), represented by Λ, can be used to assess motional averaging of the QCC.^66^ The ARF is defined as
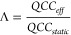
23and can be roughly equated to an order parameter. Qualitatively, the extent of deviation from the Pake doublet form correlates with the extent of the motion. The detailed nature of local molecular motions, including the direction and amplitude of the reorientational motion, can be determined by simulations.

### 3.1 Analysis of Base Line Shapes

Overall, the line shapes of the base-labeled nucleotides substantially retain the Pake doublet form, with some significant deviations. The line shapes demonstrate a distinctive slope inward at the top of the horns that is slight in the [5,6-^2^H]-C6 and [5,6-^2^H]-C8 line shapes, slightly more noticeable in the [8-^2^H]-A4 and [6-^2^H]-T9 line shapes, and very pronounced in the [8-^2^H]-G5 and [8-^2^H]-G7 line shapes. Quantification of the line shapes (Table 2) shows that there is very slight narrowing of the horns for all sites. What varies significantly is the height of the center of the line shape, which is approximately 30% for all pyrimidine bases, but is around 20% for the purines within the recognition sequence and 44% for the A4, which flanks the recognition sequence. This implies differential motional averaging in the purine bases within and outside of the recognition sequence with motional averaging being diminished in the interior purines.

**Table 2:** Measured Key Features of the Seven Experimental Line Shapes Shown in Figure 4

label site	horn width (kHz)	QCC_eff_ (kHz)	Λ	center height (% of total height)	full width at half-max. (kHz)
A4	118 ± 2	157 ± 2	0.90	44%	135 ± 2
G5	122 ± 2	162 ± 2	0.93	20%	140 ± 2
C6	122 ± 2	162 ± 2	0.93	31%	135 ± 2
G7	122 ± 2	162 ± 2	0.93	22%	142 ± 2
C8	121 ± 2	162 ± 2	0.93	34%	134 ± 2
T9	118 ± 2	158 ± 2	0.91	35%	135 ± 2
5mC6	120 ± 2	159 ± 2	0.91	30%	135 ± 2

The ⟨*T*_1*Z*_⟩ and ⟨*T*_1*Q*_⟩ values are also indicative of slower, small amplitude motions for the bases, with the C8 base standing out with the longest ⟨*T*_1*Z*_⟩ (244 ± 18 ms) and ⟨*T*_1*Q*_⟩ (195 ± 20 ms). The purine base relaxation times follow a similar trend as that observed in the line shapes, with the G5 and G7 having comparable, significantly longer ⟨*T*_1*Z*_⟩ and ⟨*T*_1*Q*_⟩ values (∼150 ms) than the A4 (∼100 ms), indicating more mobility in the A4. Interestingly, C6 and T9 have ⟨*T*_1*Z*_⟩ and ⟨*T*_1*Q*_⟩ values comparable to each other and to the interior purines (∼150 ms). The relaxation and line shape data for C8, which is the nontarget deoxycytidine within the recognition sequence, clearly shows the least indication of dynamic averaging, while the line shapes and relaxation data for the other bases within and flanking the 5′-GCGC-3′ sequence show varying indicators of dynamic averaging.

As mentioned above, it was assumed that the bases were undergoing a diffusive DNA breathing motion. The most logical form for *U*(ϕ) in this case is a harmonic potential:
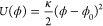
28where ϕ_0_ is the point on the path with the minimum energy, assumed here to be the stacked, base-paired position, and κ is proportional to the restoring force of the hydrogen bonds. This motion, along with the global helical rotation, sufficiently fit the line shape and ⟨*T*_1*Z*_⟩ data, but not the ⟨*T*_1*Q*_⟩ data. The addition of a fast, small amplitude base libration had a significant effect on the ⟨*T*_1*Z*_⟩ and ⟨*T*_1*Q*_⟩ simulations, enabling successful fits of all of the data. Notably, the line shapes alone could be fit equally well with significantly different parameters defining the breathing motion, but the ⟨*T*_1*Z*_⟩ and ⟨*T*_1*Q*_⟩ simulations responded uniquely to the added librational motion when the breathing motion was defined by a force constant of 0.5 *k*_B_*T*, angular excursions of 10–12°, and diffusion rates on the order of 10^6^ Hz, increasing confidence in these results.

The parameters describing the best-fit line shapes are shown in Table 3. The breathing motion of all line shapes was simulated using the aforementioned harmonic potential with a force constant of 0.5*k*_B_*T*. Additionally, the breathing of all but the C8 line shape were simulated best by an angular excursion between 12° and 15° and a jump rate between 3 × 10^6^ and 7 × 10^6^ Hz. The C8 required a significantly smaller angular excursion of 9.5° with a rate of 3 × 10^6^ Hz, at the slow end of the range of the other sites. For all sites, the base libration was simulated by an amplitude of either 5° or 6° with a rate between 1 × 10^8^ and 1 × 10^9^ Hz.

**Table 3:** Parameter Values Used to Simulate Each of the Seven Line Shapes in Figure 4^a^

label site	κ (*k*_B_*T*)	angular excursion	jump rate (Hz)	base libration	jump rate (Hz)
A4	0.5	13°	7 × 10^6^	6°	1 × 10^8^
G5	0.5	15°	3 × 10^6^	5°	4 × 10^8^
C6	0.5	12°	5 × 10^6^	6°	1 × 10^9^
G7	0.5	15°	3 × 10^6^	5°	4 × 10^8^
C8	0.5	9.5°	2.5 × 10^6^	6°	1 × 10^8^
T9	0.5	12.5°	4 × 10^6^	5°	1 × 10^8^
5mC6	0.5	12°	5 × 10^6^	6°	1 × 10^9^

a The potential used for these simulations was .

### 3.2 Impact of C6 Methylation on Base Dynamics

There is not a significant change in the line shape of C6 upon methylation (Figure 4), only a slight increase in definition of the shoulders and a small decrease in the height of the center of the line shape, indicating that this site experiences negligible changes in motion upon methylation. Additionally, the ⟨*T*_1*Z*_⟩ values are equivalent, within error (144 ± 7 and 150 ± 5 ms) between the unmethylated and methylated C6. Though the extent of the dynamics of C6 is not clear from a qualitative analysis of the line shapes and relaxation data, what does remain clear is the minimal loss of mobility associated with the methylation of C6.

The unmethylated C6 line shape was quite similar to the other bases and was best fit using a harmonic potential with a force constant of 0.5*k*_B_*T* an angular excursion of 12° and a rate of 5 × 10^6^ Hz for the breathing motion and an amplitude of 6° and a rate of 1 × 10^9^ Hz for the base libration. Upon methylation, the C6 line shape is best modeled by the same parameters. These fits support the conclusions drawn from the qualitative analysis, namely, that if any change in conformational flexibility upon methylation of C6 occurs, it is negligible.

## 4 Discussion

The sequence-specificity of DNA recognition and in particular, base flipping, by the *Hha*I methyltransferase has been extensively studied: structurally,^22,32,36,72−74^ computationally,^20,26,27,30−32,44,60,75−78^ biochemically,^23,25,38,79^ and by dynamic spectroscopy.^22,46,52,80,81^ Base pair opening has previously been shown to be sequence-dependent.^62^ Additionally, GC base pairs are known to experience longer lifetimes (10–50 ms) than AT base pairs (1–5 ms),^57−59^ with significantly higher base pair opening rates observed in GC tracts^21^ and significantly lower rates in AT tracts.^63^ Molecular dynamics simulations of base pair opening indicate that the purine bases have significantly lower barriers to base pair opening than their pyrimidine base pairs.^60^ Here, the line shapes and relaxation times indicate significant variability in the dynamics of the bases of the DNA dodecamer, especially between purines and pyrimidines within and flanking the recognition site (Tables 1 and 2).

Recognition processes are very complex, and it is not surprising that dynamics may be one of many factors that contribute, as suggested by the work presented here. When compared to the flanking sequence, the relaxation times throughout the recognition site are quite consistent, with the exception of C8, which has the slowest relaxation times of the entire sequence (and of all of the pyrimidines studied). The unique relaxation time of the C8 is particularly interesting in light of its known complete inhibition of cleavage by the *Hha*I endonuclease when methylated.^82^ The limited degree of dynamic variability along the DNA sequence indicated by the line shapes and relaxation times is supported by the simulations, with the target C6 (and A4 and T9) experiencing a slightly more restricted motion as compared to the G5 and G7 and with C8 experiencing the most restricted motion (Table 3).

Other techniques have been used to probe the dynamics of DNA, primarily high-resolution by monitoring the relaxation of the ^13^C spins by the use of solution NMR.^83−89^ Previous solution-state ^13^C relaxation studies of the DNA sequence studied here have suggested that internal motions across the *Hha*I methylation target sequence are uniform and unremarkable,^81^ as was also reported for the Dickerson dodecamer.^90^ In contrast, the solid-state data clearly demonstrate that dynamics of the bases differ along the *Hha*I recognition sequence, particularly at the C8.

It has been previously established that DNA motions in the solid state become essentially solution-like once the DNA becomes fully hydrated, which occurs at levels below those used in the present investigation.^91,92^ Thus, it is very unlikely that the differences between the two techniques can be attributed to different DNA dynamics. The motions responsible for modulation of the solid-state line shapes likely occur on time scales that are not easily discernible in solution NMR relaxation experiments, namely, the microsecond–nanosecond time scale. Although residual dipolar couplings may probe molecular dynamics in this regime, they can only give information about amplitudes of motion and not rates.^93,94^

There are two competing theories on how base flipping is initiated (active versus passive). In active flipping, the protein binds to typical B-form DNA and then breaks the necessary hydrogen bonds and alters the local backbone structure to extrude the target nucleotide. In passive flipping, the enzyme simply senses the base while it exists in the flipped configuration, captures it, and inserts it properly into the binding pocket. The growing consensus supports active flipping as the most likely model, with more supporting evidence for active^26,38,95^ than passive flipping.^39^ The results of this work provide additional support for the active flipping model with no evidence of a large amplitude breathing motion for the target C6 base. However, the C6 base is less energetically restricted than the C8 which combined with the increased flexibility previously observed in the furanose ring^51^ and phosphodiester backbone^47^ indicates a decreased barrier to base flipping.

The impact that methylation exerts on [5′-GCGC-3′]_2_ sequences has been studied structurally,^96−99^ biochemically,^100,101^ and by dynamic spectroscopy.^52,102^ Given the impact that methylation of the target base in the *Hha*I binding site has on both cleavage by the endonuclease and binding of the Mtase,^37^ it may seem natural to expect some effect on the dynamics of the base. Therefore, it is most surprising that the experimental results and simulations indicate no effect of methylation on the base of the C6 nucleotide within experimental error. In contrast, we have previously shown that the dynamics of both the phosphodiester backbone and the furanose ring are both reduced by methylation, with the impact on the backbone being much more substantial.^47,51^

Of some concern is how representative the dodecamer studied here is of sequences within longer polynucleotides. Recently, FRET, SAXS, AFM, and computational studies have shown that short and intermediate length DNAs (<100 base pairs) exhibit an enhanced flexibility,^103−105^ when compared to average measurements of longer oligonucleotides.^104−106^ These studies focus on bulk flexibility of DNA and do not look at local dynamics, but imino proton exchange studies have also shown the lifetimes of central base pairs to decrease with the length of the oligomer.^107^ However, our analysis focuses on variability along the sequence and, therefore, should not be affected by the length of the DNA.

## 5 Conclusions

Here we have collected line shape data and measured ⟨*T*_1*Z*_⟩ and ⟨*T*_1*Q*_⟩ for the bases of the nucleotides within the DNA recognition site for the *Hha*I methyltransferase. A qualitative analysis of both the line shapes and the relaxation data indicates that C8 is significantly less mobile than the other bases within the recognition sequence. While both deoxycytidine residues display dynamic behavior within their furanose rings,^51^ only the C8 position has significantly restricted motional averaging in its base. This suggests that the dynamics observed at this position, which is not the methylation target of the *Hha*I methyltransferase, might play some sort of role in the restriction-modification mechanism.

The local dynamics may play an important role in the initiation of base flipping, with the target site showing more conformational flexibility in the base, furanose ring, and phosphodiester backbone than any other position within the binding site, which effectively lowers the barrier for cytosine base extrusion. Our simulations show that the bases of the nucleotides in [5′-GCG↓C-3′]_2_ demonstrate energetically restricted motion (Table 3). Therefore, it is not surprising that the structural flexibility of the DNA backbone near the methylation site is reflected in a similar mobility of the furanose rings,^47,51^ since here, the furanose ring must act as the intermediary between the backbone and the base with the former demonstrating significantly more dynamics than the latter.
